# Development of a low-penetrance mitosis instead of meiosis system in tomato

**DOI:** 10.1186/s43897-025-00207-6

**Published:** 2026-04-01

**Authors:** Tong Zhao, Yi Zhang, Jiayao Wu, Iqbal Hussain, Weiqiang Li, Fan Ping, Kaiwen Liu, Changtian Pan, Xiaolin Yu

**Affiliations:** 1 Department of Horticulture, College of Agriculture and Biotechnology, Zhejiang University, Hangzhou, 310058 China; 2 Laboratory of Horticultural Plant Growth & Quality Regulation, Ministry of Agriculture, Hangzhou, 310058 China; 3 Group of Vegetable Breeding, Hainan Institute of Zhejiang University, Sanya, 572025 China

Tomato, a representative species of the Solanaceae family, ranks as the second vegetable crop globally; conventional crossbreeding remains the principal approach for improving tomato cultivars currently (Sharma et al. 2019). However, producing hybrid tomato seeds remains a time-consuming, labor-intensive, and costly process on an annual basis. Apomixis—a process that avoids meiosis and fertilization—results in cloned seeds genetically identical to the female parent, offering substantial potential for stabilizing heterosis (Xiong et al. 2023). The *MiMe* (mitosis instead of meiosis) system, successfully established in *Arabidopsis*, rice, and tomato, is gaining widespread application (d’Erfurth et al. 2009; Mieulet et al. 2016; Wang et al. 2024). A critical step in the *MiMe* system involves omitting the second meiotic division to generate unreduced gametes. In *Arabidopsis* and rice, this is achieved by knocking out the *OSD1* gene, while in tomato, a similar effect is induced by the knockout of *SlTAM*. In model species like *Arabidopsis* and rice, two homologous genes—*OSD1* and *UVI4*—regulate the cell cycle, with *OSD1* specifically influencing germ cell cycle regulation and *UVI4* controlling somatic cell cycles (Hase et al. 2006; Cromer et al. 2012). However, crops such as pepper, tomato, potato, and watermelon possess only a single homolog of OSD1. Recent studies have shown that knockout of *ClOSD1* in watermelon induces the doubling of both somatic and germ cells, highlighting the challenges associated with utilizing *OSD1* for the construction of *MiMe* mutants in these species (Pang et al. 2025). Here, we constructed a weak allelic mutant of *Slosd1* in tomato, which produced a small number of 2n pollen grains while maintaining the diploid state of somatic cells. When *SlSPO11-1*, *SlREC8*, and *SlOSD1* were simultaneously knocked out, the resulting triple knockout mutant exhibited a phenotype resembling mitosis instead of meiosis, with lower penetrance.

We employed CRISPR/Cas9 genome editing to generate single and triple mutants of the tomato ‘Micro Tom’ cultivar by targeting the *SlSPO11-1*, *SlREC8*, and *SlOSD1* genes through four independent transformation experiments (Figs. S1–S4). Among these mutants, the SlSPO11-1 and SlREC8 proteins exhibited frameshift mutations, resulting in premature protein translation termination, while SlOSD1 showed only weak allelic mutations due to partial amino acid deletions (Figs. S2 and S4). No abnormalities were observed during the vegetative growth of *Slspo11-1* and *Slrec8* mutants (Fig. 1A). As anticipated, pollen viability significantly reduced in both, with nearly 100% inactivity (Fig. 1B–C), and shrunken pollen grains were evident under scanning electron microscopy (Fig. 1B). However, *Slosd1* mutants also exhibited notable pollen abortion, with numerous shriveled and shrinking pollen grains observed (Fig. 1B). Additionally, pollen viability varied among different *Slosd1* lines; only lines #11 and #2 produced fruit, though fruit size was significantly smaller and contained fewer seeds than wild-type plants (Fig. S5). Among the *SlMiMe* mutants, pollen viability showed considerable variation. Lines #12 and #20 were completely sterile, while #2, #3, and #21 exhibited pollen viability ranging from 10.73% to 30.66% (Fig. S5A–B). The morphology of viable pollen in triple mutants is comparable to that of wild-type plants (Fig. 1B). The *SlMiMe*-#2 and #3 produced fruits, though these were smaller in size with fewer seeds (Fig. S5C–F).

**Fig. 1:**
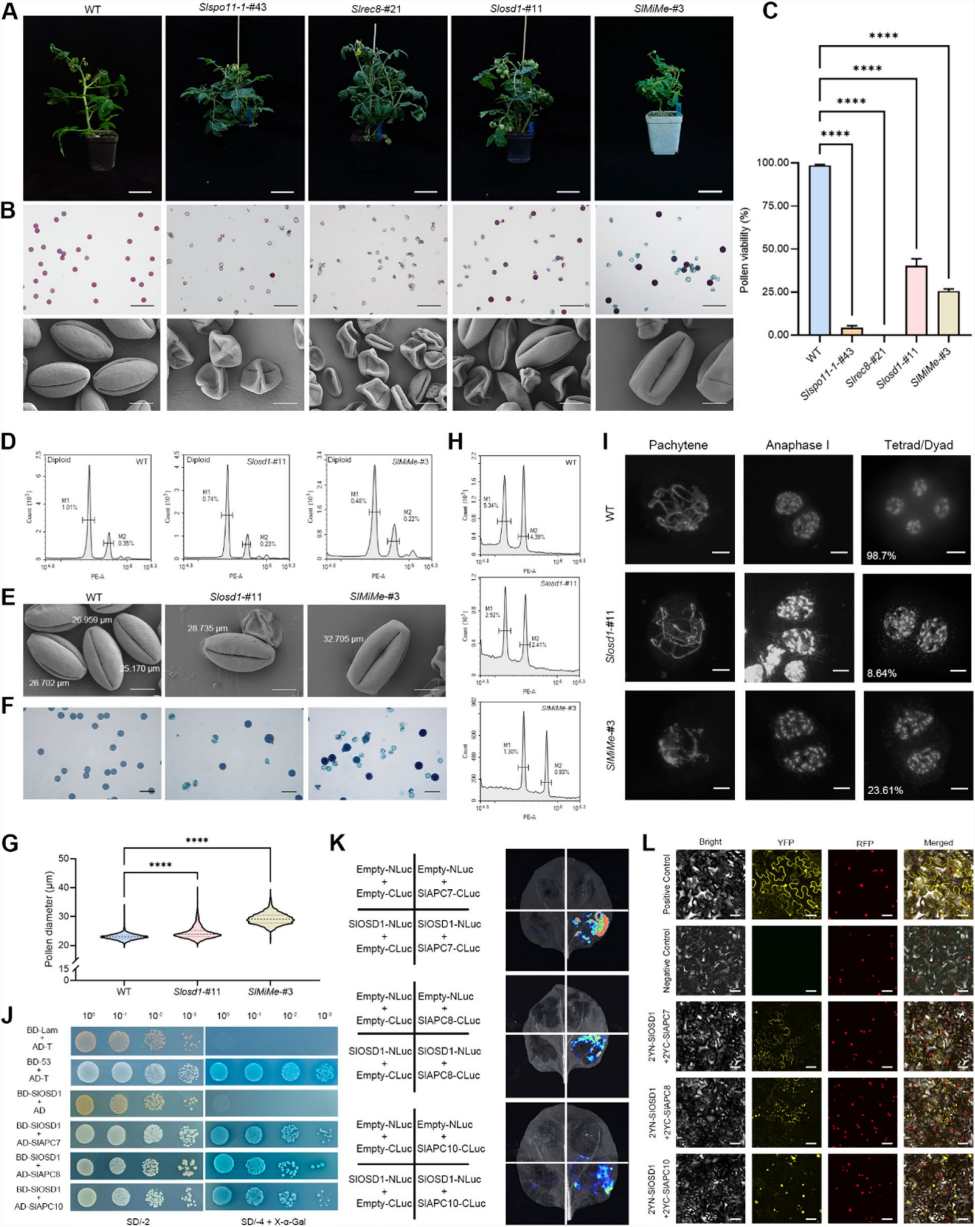
Simultaneous editing of *SlSPO11-1*, *SlREC8*, and *SlOSD1* led to the emergence of a mitosis instead of meiosis phenotype with low penetrance. **A** Phenotypes of *Slspo11-1*, *Slrec8*, *Slosd1*, and *SlMiMe* tomato mutants. Bars, 5 cm. **B** Pollen vitality examination and scanning electron microscopy observation. Row 1, bars, 100 μm. Row 2, bars, 10 μm. **C** Pollen viability of mature pollen grains based on Alexander dye staining assay in (**B**). Data were presented as means ± SD (*n* = 3). Over 500 pollen grains were analyzed in each replication. Asterisks (****p < 0.0001) represent statistically significant differences between samples after one-way ANOVA and Dunnett's multiple comparison tests. ns denoted no significant difference. **D** Ploidy analysis of representative T_0_ generation *Slosd1* and *SlMiMe* mutants. **E** Scanning electron microscopy images of enlarged pollen grains produced by *Slosd1* and *SlMiMe* diploid mutants. Bars, 10 μm. **F** Alexander dye staining images of enlarged pollen grains produced by *Slosd1* and *SlMiMe* diploid mutants. Bars, 50 μm. **G** Pollen diameter of *Slosd1* and *SlMiMe* diploid mutants. The diameters of viable pollen from the mutants were measured using ImageJ, with each group comprising measurements of over 2,500 pollen grains. Asterisks (****p < 0.0001) represent statistically significant differences between samples after one-way ANOVA and Dunnett's multiple comparison tests. **H** Pollen ploidy of wild type, *Slosd1*, and *SlMiMe* diploid mutants was detected by flow cytometry. WT and *Slosd1*-#11 groups underwent three biological replicates, whereas due to the limited pollen available, only one biological replicate was conducted for *SlMiMe*-#3. **I** Meiotic chromosome behaviors of wild type, *Slosd1, and SlMiMe* tomato mutants. Bars, 10 μm. **J** Y2H assay showing SlOSD1 interaction with SlAPC7/8/10. Co-transformed yeast cells with the indicated plasmids were plated on selection medium SD/–2 and SD/–4 + X-α-Gal. **K** Luciferase complementation imaging assays to validate the interaction between SlOSD1 and SlAPC7/8/10. **L** The BiFC assays detected interactions between SlOSD1 and SlAPC7/8/10. SlOSD1 was fused to the N-terminal region of YFP, while SlAPC7/8/10 were fused to the C-terminal region of YFP. The nuclei positions are indicated by the H2B–RFP marker. Bars, 5 μm

Flow cytometry analysis revealed that among the three T_0_ *Slosd1* lines, #11 and #2 were diploid, while #3 was tetraploid (Figs. 1D, S6A). In the five T_0_ *SlMiMe* mutants, lines #2, #3, #12, and #21 were diploid, and line #20 was tetraploid (Figs. 1D, S6B). Diploid lines were selected for further analyses. SEM analysis and Alexander staining revealed the presence of enlarged pollen grains in both *Slosd1* and *SlMiMe* mutants (Fig. 1E–F). To minimize errors from non-viable pollen, we measured the diameters of only viable pollen grains using ImageJ. Both mutants produced significantly larger pollen than those of the wild-type (Fig. 1G). Specifically, in the *Slosd1*-#11 line, 6.29% (*n* = 2576) of viable pollen grains were classified as large (≥ 28.15 μm). In the *SlMiMe* mutant #3 line, 69.36% (*n* = 2523) of viable pollen grains showed increased size.

To further ascertain whether *Slosd1* and *SlMiMe* mutants produce 2n pollen, flow cytometry was employed to analyze mature pollen ploidy. In wild-type plants, two peaks were observed in the pollen profile—haploid (vegetative nucleus) and diploid (generative nucleus) —consistent with previous study (Bino et al. 1990). However, in the *Slosd1* mutant, a tetraploid peak was absent, likely due to the low frequency of 2n pollen falling below the detection threshold of the instrument. In contrast, the *SlMiMe* mutants displayed both diploid and tetraploid peaks, confirming the presence of 2n pollen (Fig. 1H).

Due to the significant reduction in pollen viability and limited seed production in the *Slosd1*-#11 mutant, we selected the *Slosd1*-#2 mutant for progeny ploidy analysis. This line carried a large-segment knockout with a 72 bp deletion, exhibited a homozygous mutation in all T_1_ progeny. Progeny ploidy analysis of the *Slosd1*-#2 mutant revealed among 18 T_1_ generation plants, one was tetraploid plant and the rest were diploid (Fig. S7A–C). Additionally, six T_1_ plants from the *SlMiMe*-#3 line were successfully grown, and flow cytometry confirmed that all were tetraploid (Fig. S7D–F). These findings further confirmed the existence of 2n gametes.

To further examine meiotic progression in *Slosd1* and *SlMiMe* mutants, we employed DAPI staining. In wild-type tomato plants, homologous chromosomes aligned and paired during pachytene, with 98.7% of cells successfully forming tetrads following two meiotic divisions (*n* = 76, Fig. 1I). The chromosome morphology observation in the *Slosd1* diploid mutants during meiosis revealed the presence of dyads, with 8.64% failing to undergo the second meiotic division and directly forming gametes, while the remaining cells formed tetrads (*n* = 81, Fig. 1I). Similarly, 23.61% of the male meiotic products of *SlMiMe* diploid mutants omitted the second meiotic division, forming diploid dyads and exhibiting a mitosis-like phenotype (*n* = 144, Fig. 1I).

In *Arabidopsis*, OSD1 facilitates meiotic progression by inhibiting the activity of the anaphase-promoting complex/cyclosome (APC/C). Notably, OSD1 does not associate with any core APC/C subunits but instead interacts with several APC/C activators (Iwata et al. 2011; Cromer et al. 2012). Conversely, our previous study in pepper showed that CaOSD1 directly interacts with the core APC/C subunits CaAPC7/8/10 (Zhao et al. 2025). Given the high sequence similarity of OSD1 proteins between tomato and pepper, we initially investigated the interaction between SlOSD1 and SlAPC7/8/10 in tomato using yeast two-hybrid (Y2H) assays. The results indicated that SlOSD1 exhibits no self-activation and can interact with SlAPC7/8/10 (Fig. 1J). These interactions were further confirmed by luciferase complementation imaging (LCI) and bimolecular fluorescence complementation (BiFC) assays (Fig. 1K–L). Collectively, these findings suggested that OSD1 proteins in Solanaceae species may function through a regulatory mechanism distinct from that in *Arabidopsis*.

Previous studies on tomato have failed to generate a diploid null allelic mutant of *Slosd1*. Wang et al. (2024) only achieved tetraploid chimeric mutants, while Di et al. (2022) produced diploid mutants with partial loss-of-function. In this study, *SlOSD1* knockout also induced the T_0_ tetraploid plants. The *Slosd1* weak allelic mutant we obtained is more similar to the one described by Di et al. (2022), as both mutants exhibit only partial amino acid deletions. Differences in phenotype may be due to variation in sgRNA target sites and the types of mutations induced. Structural predictions using AlphaFold 3 revealed that even minor amino acid changes significantly altered the overall structure of SlOSD1 protein (Fig. S8). This suggests that a complete deletion of the *SlOSD1* gene would significantly impact both somatic and germ cells in tomato. In contrast, the weak allelic mutants may partially mitigate this phenotype, leading to the generation of diploid mutants. Further investigations are necessary to clarify how *OSD1* deficiency leads to the induction of plants with varying ploidy levels and to explore its conservation of biological function across plant species.

In summary, our study found that the *Slosd1* mutant with partial loss of function produces a small number of 2n pollen grains while retaining a diploid state in somatic cells. Compared with results of *Arabidopsis* (d’Erfurth et al. 2009) and rice (Mieulet et al. 2016), the generation of *SlMiMe* mutants with null alleles in *SlSPO11-1* and *SlREC8*, in combination with weak alleles in *SlOSD1*, led to a phenotype characterized by mitosis instead of meiosis, though with low penetrance. These results hint that the biological functions of *SlSPO11-1*, *SlREC8*, and *SlOSD1* are generally conserved across species, but there are also some functional differentiations. Combining mutations in these three genes significantly reduces seed production, presenting a challenge in the construction of a synthetic apomixis system in tomato. Moreover, the interaction between SlOSD1 and SlAPC7/8/10, suggesting that OSD1 in Solanaceae operates via distinct regulatory pathways. Overall, our research findings provide the theoretical foundation and useful clues for the development of a synthetic apomixis system in tomato and advancing ploidy-based breeding strategies.

## Supplementary Information

Supplementary Material 1.

## Data Availability

The datasets used during the current study are available from the corresponding author upon reasonable request.
