# Supplementary material for: Development of a low-penetrance mitosis instead of meiosis system in tomato
Source: Mol Hortic. 2026 Apr 1;6:38. doi: 10.1186/s43897-025-00207-6 (PMC13041034; doi:10.1186/s43897-025-00207-6)
Supplement: Supplementary file 1 — Supplementary Material 1. [file 43897_2025_207_MOESM1_ESM.docx]

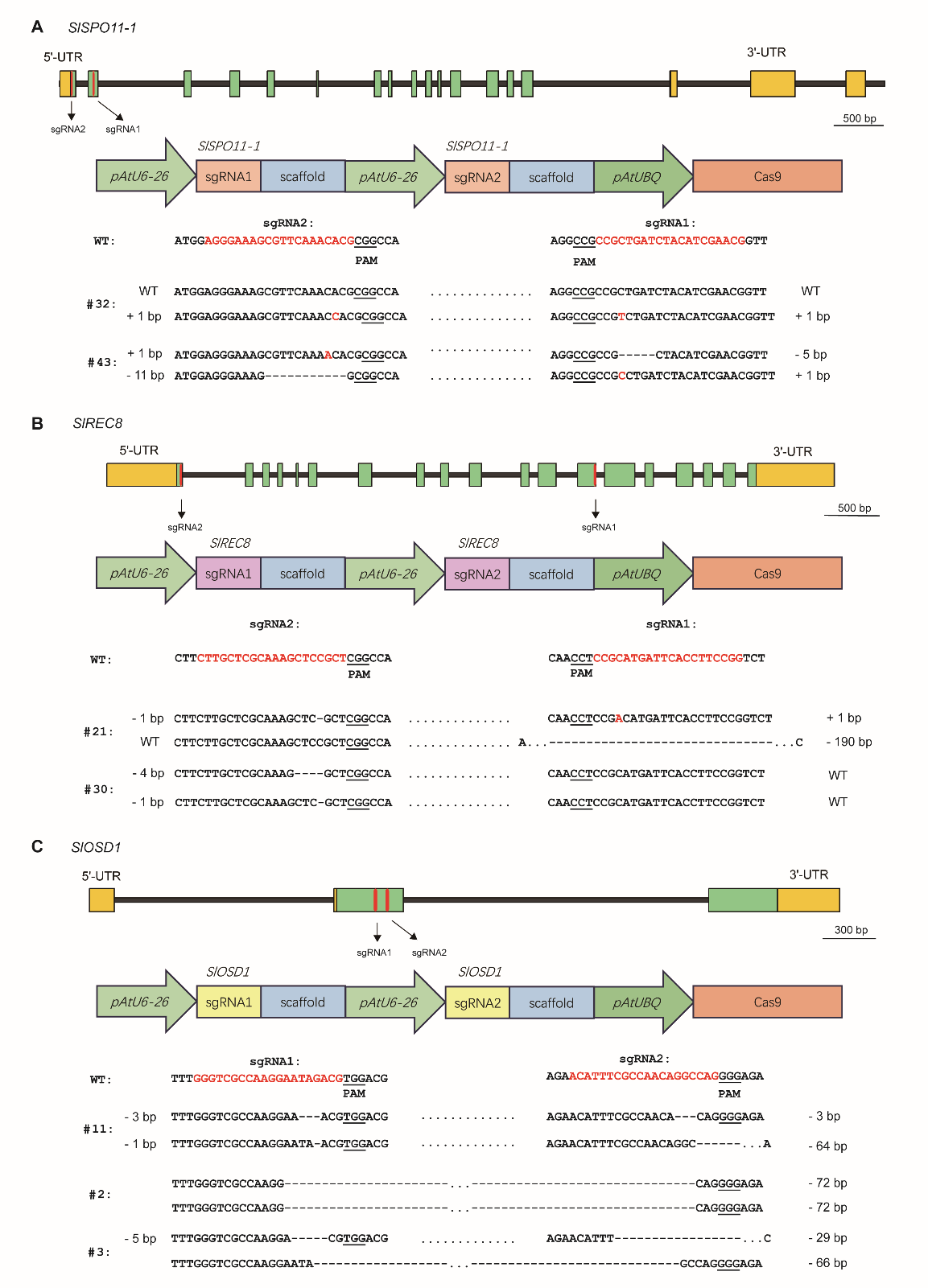


**Fig. S1.** Schematic representation of the gene structures of *SlSPO11-1*, *SlREC8*, and *SlOSD1*, along with CRISPR/Cas9-induced mutations at the target sites. Black lines denote introns, green boxes represent exons, and yellow boxes indicate untranslated regions (UTRs). Additionally, a red box highlights the location of the designed single guide RNA (sgRNA). For sequences around sgRNA in the WT, the red letters represent the sgRNA sequence and the underlined letters represent the protospacer-adjacent motif (PAM) sequence. In knockout lines, mutations are indicated as follows: a black dashed line ‘-’ signifies a base deletion, while a red letter indicates the inserted base.


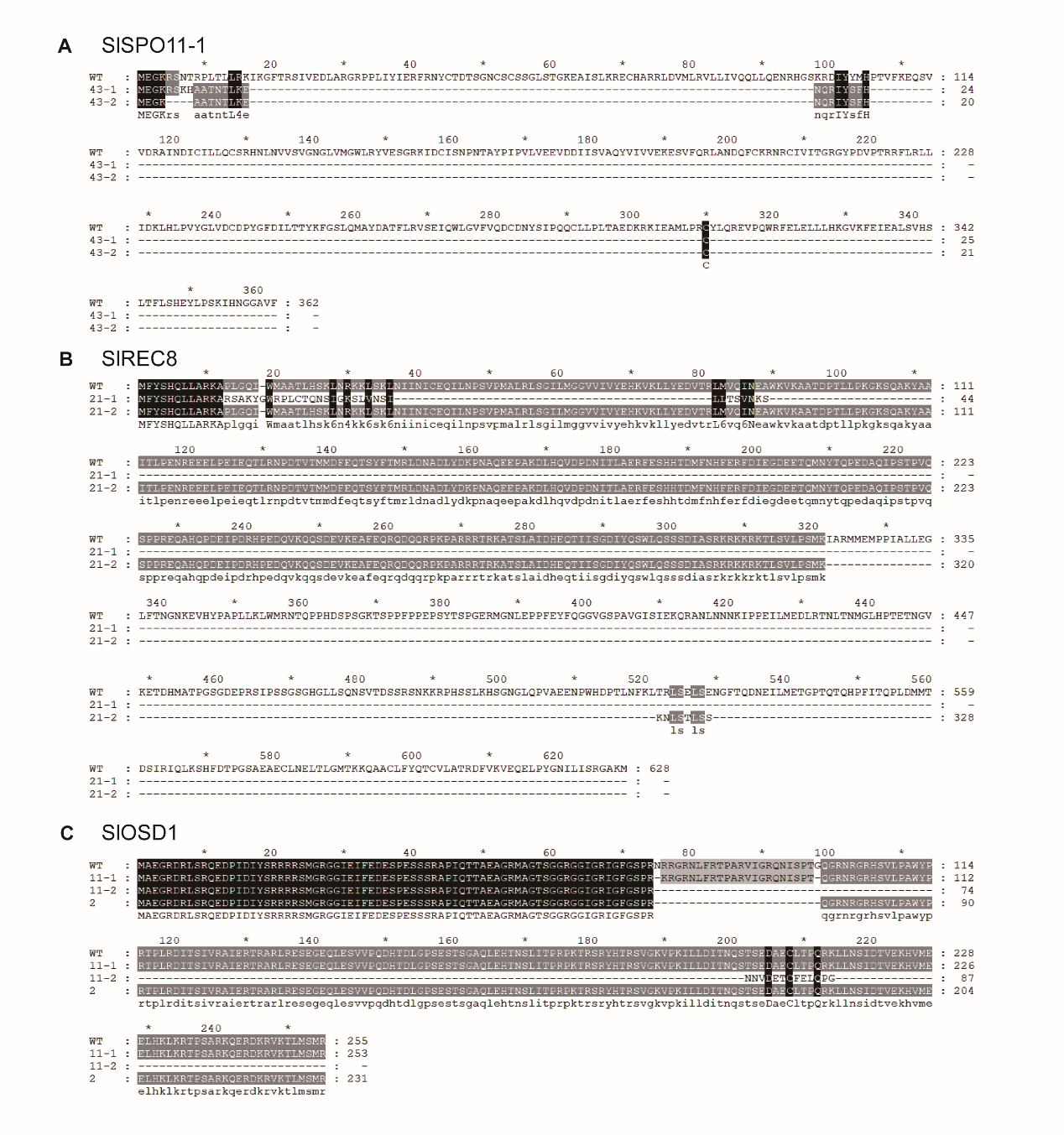


**Fig. S2** Protein sequence alignment of representative *Slspo11-1*, *Slrec8*, and *Slosd1* mutants. **A** Alignment of protein sequences between *Slspo11-1*-#43 mutants and wild-type plants. **B** Alignment of protein sequences between *Slrec8*-#21 mutants and wild-type plants. **C** Alignment of protein sequences between *Slosd1*-#11, *Slosd1*-#2 and wild-type plants. The number following each line represents the different amino acid sequences encoded by the two alleles.


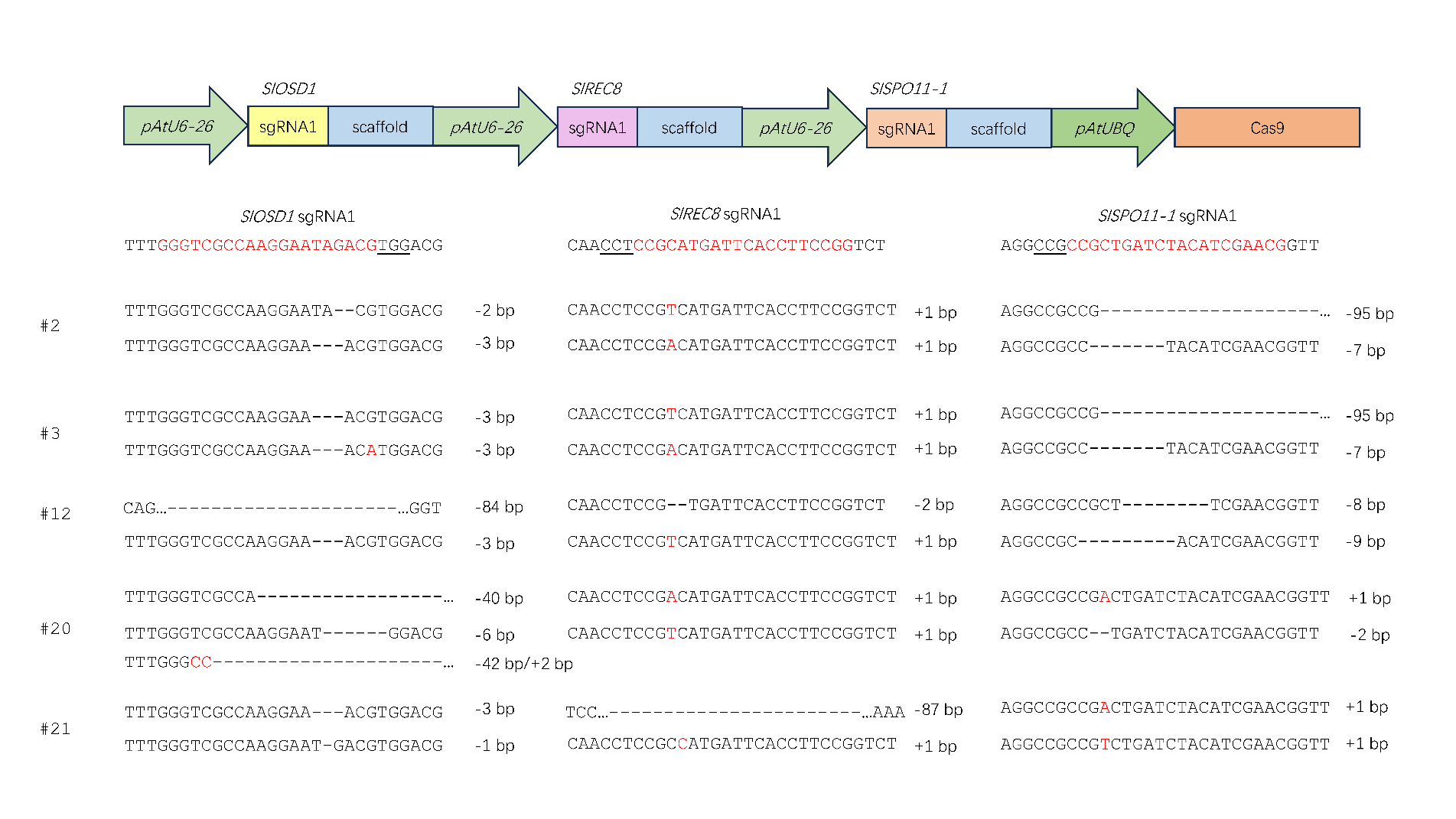


**Fig. S3** Structure of CRISPR/Cas9 vector simultaneously targeting *SlOSD1*, *SlREC8*, and *SlSPO11-1* genes and mutation sites in different lines. For sequences around sgRNA in the WT, the red letters represent the sgRNA sequence and the underlined letters represent the protospacer-adjacent motif (PAM) sequence. In knockout lines, mutations are indicated as follows: a black dashed line ‘-’ signifies a base deletion, while red letters indicate the inserted bases.


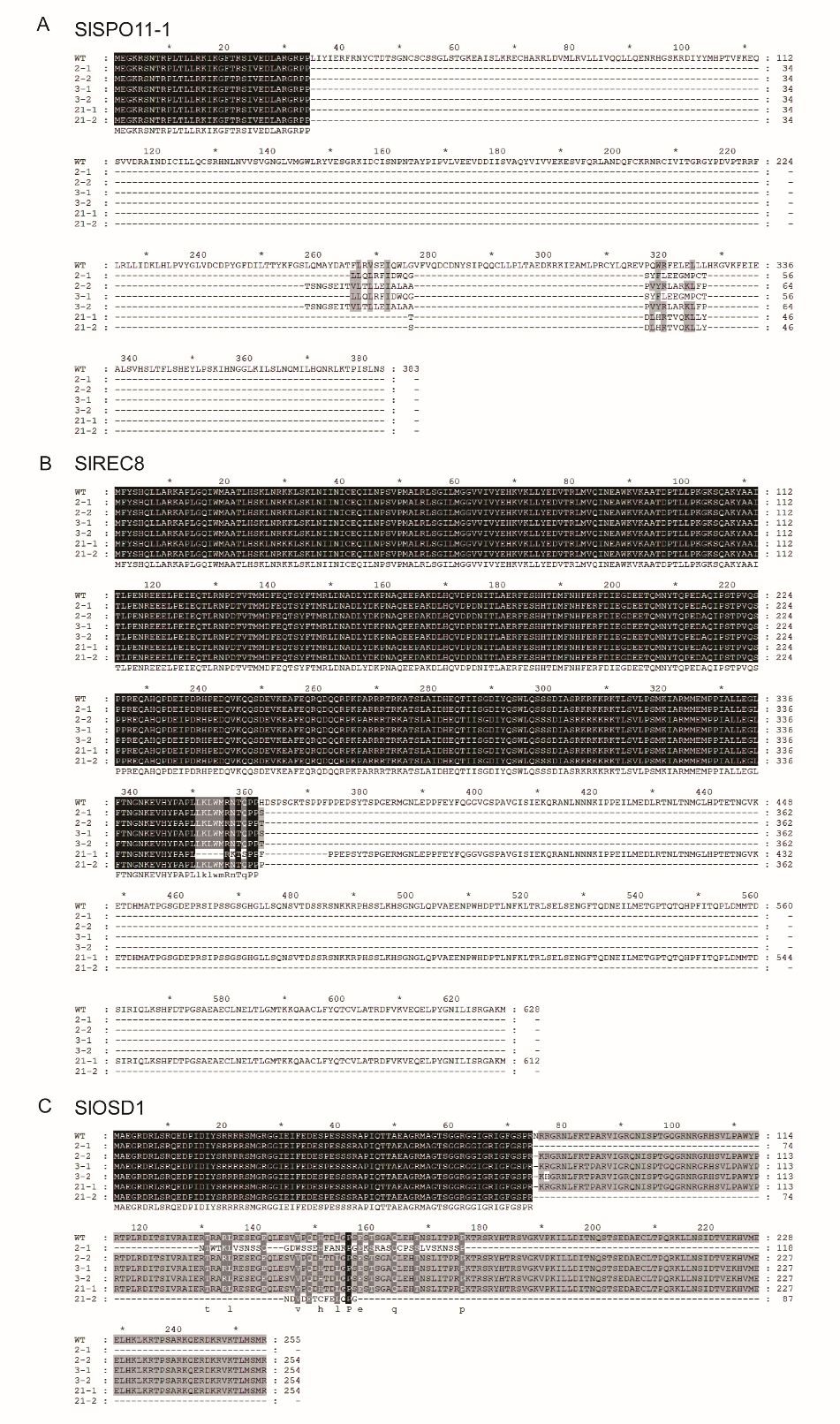


**Fig. S4** Protein sequence alignment of representative *SlMiMe* mutants. **A** Alignment of SlSPO11-1 protein sequences from *SlMiMe*-#2, #3 and #21 mutants compared with the wild-type plants. **B** Alignment of SlREC8 protein sequences from *SlMiMe*-#2, #3 and #21 mutants and wild-type plants. **C** Alignment of SlOSD1 protein sequences from *SlMiMe*-#2, #3 and #21 mutants and wild-type plants. Numbers following each sequence denote distinct amino acid variants encoded by the two alleles.


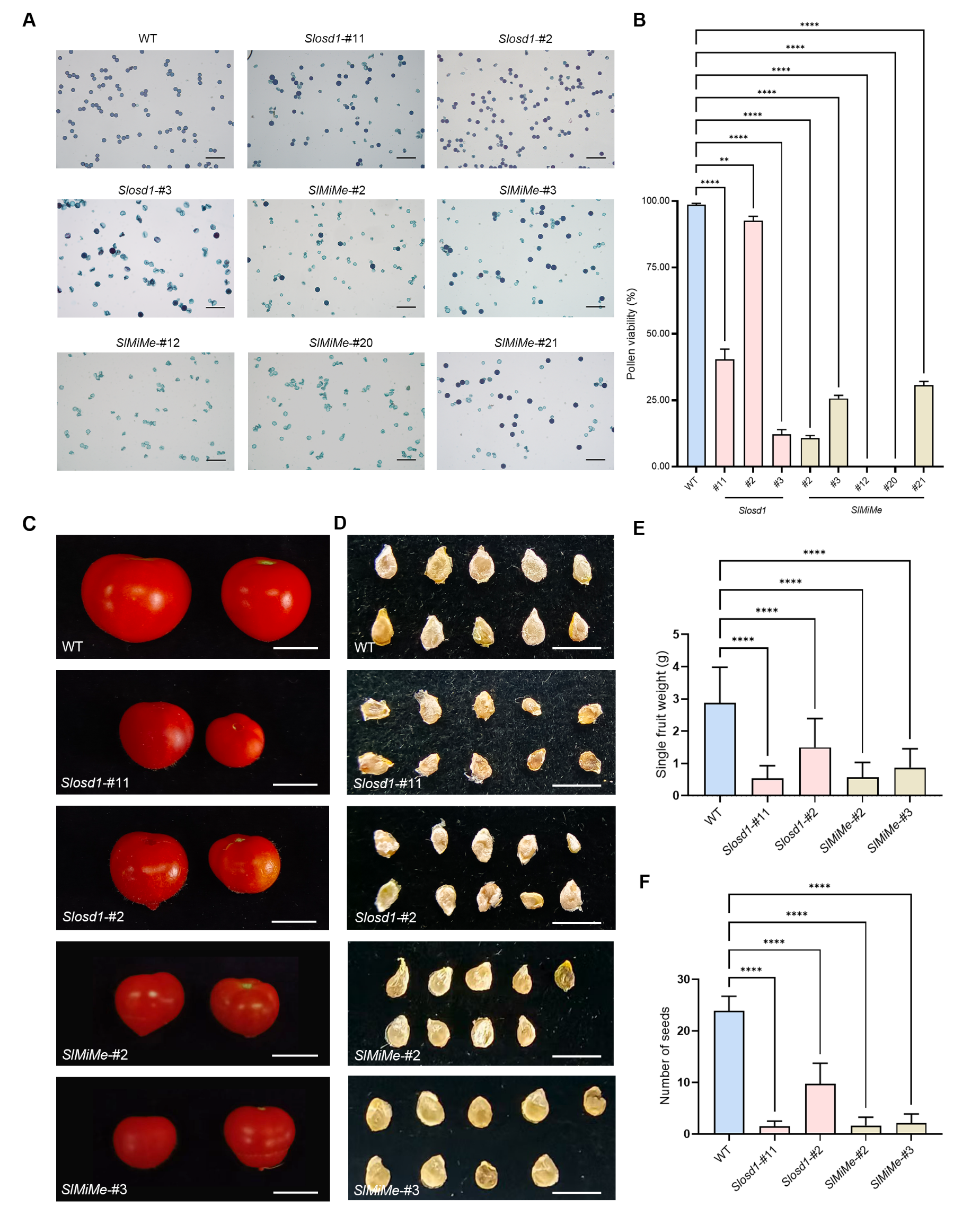


**Fig. S5** Assessment of pollen viability and evaluation of fruit traits in the *Slosd1* and *SlMiMe* mutants across different lines. **A** Alexander staining was employed to assess the pollen viability of various *Slosd1* and *SlMiMe* lines. Bars, 100 μm. **B** Statistical analysis of Alexander staining results for mature pollen grains. Data were presented as means ± SD (*n* = 3). Over 500 pollen grains were analyzed in each replication. Asterisks (***p* < 0.01, *****p* < 0.0001) represent statistically significant differences between samples after one-way ANOVA and Dunnett's multiple comparison tests. **C** Representative fruit phenotypes of wild type, *Slosd1*-#11, *Slosd1*-#2, *SlMiMe*-#2, and *SlMiMe*-#3 mutants. Bars, 1 cm. **D** Representative seed phenotypes of wild type, *Slosd1*-#11, *Slosd1*-#2, *SlMiMe*-#2, and *SlMiMe*-#3 mutants. Bars, 5 mm. **E** Statistical analysis of single fruit weight in wild type, *Slosd1*-#11, *Slosd1*-#2, *SlMiMe*-#2, and *SlMiMe*-#3 mutants. Data were presented as means ± SD (n≥10). Asterisks (*****p* < 0.0001) represent statistically significant differences between samples after one-way ANOVA and Dunnett's multiple comparison tests. **F** Statistical analysis of seed counts per fruit in wild type, *Slosd1*-#11, *Slosd1*-#2, *SlMiMe*-#2, and *SlMiMe*-#3 mutants. Data were presented as means ± SD (n≥10). Asterisks (*****p* < 0.0001) represent statistically significant differences between samples after one-way ANOVA and Dunnett's multiple comparison tests.


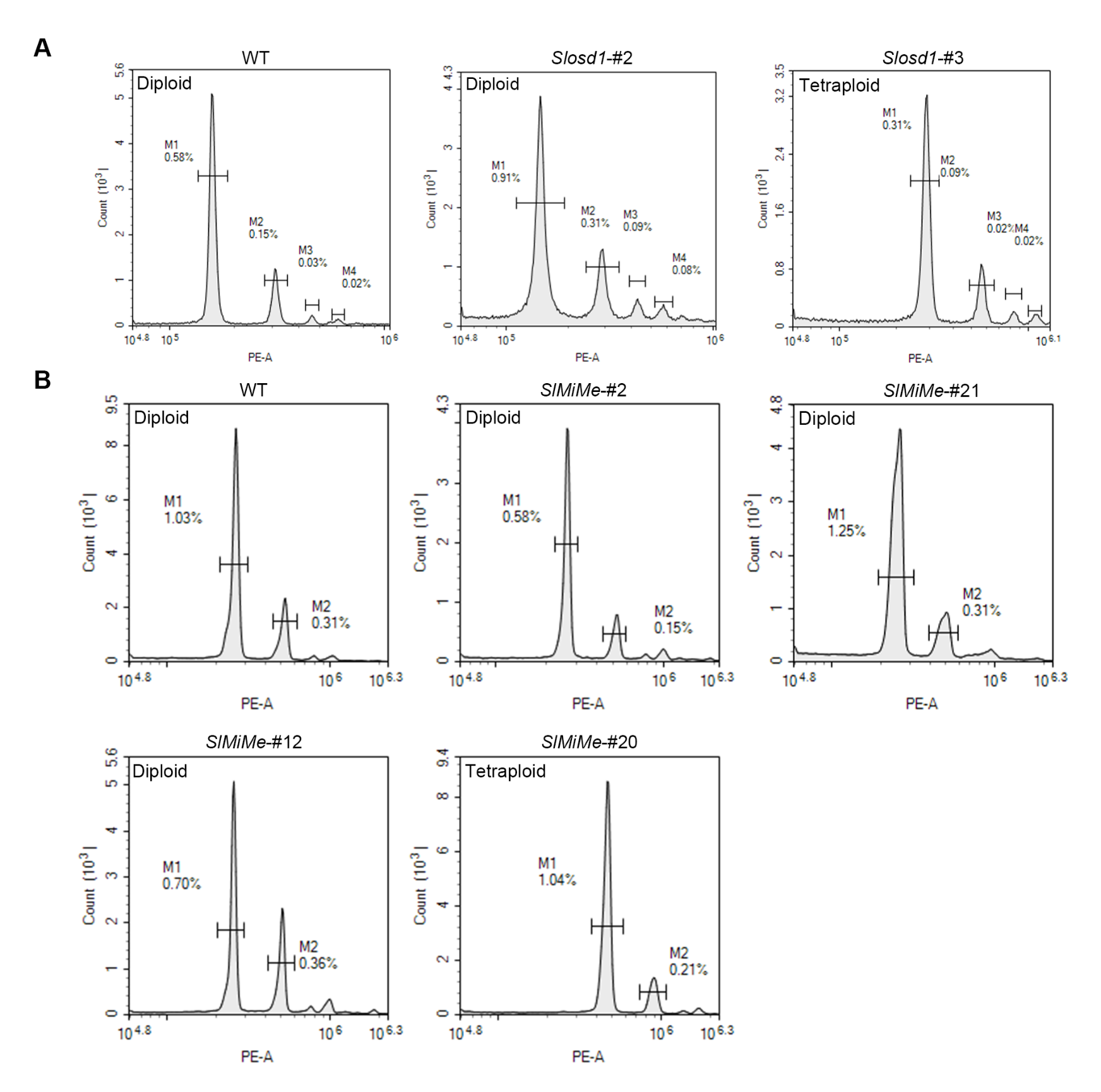


**Fig. S6** Determination of the ploidy levels in *Slosd1* and *SlMiMe* mutants across various lines. **A** Ploidy analysis of *Slosd1* mutant plants revealed that lines #2 is diploid, while line #3 is tetraploid. **B** Ploidy analysis of *SlMiMe* mutant plants indicated that lines #2, #21, and #12 are diploid, whereas line #20 is tetraploid.


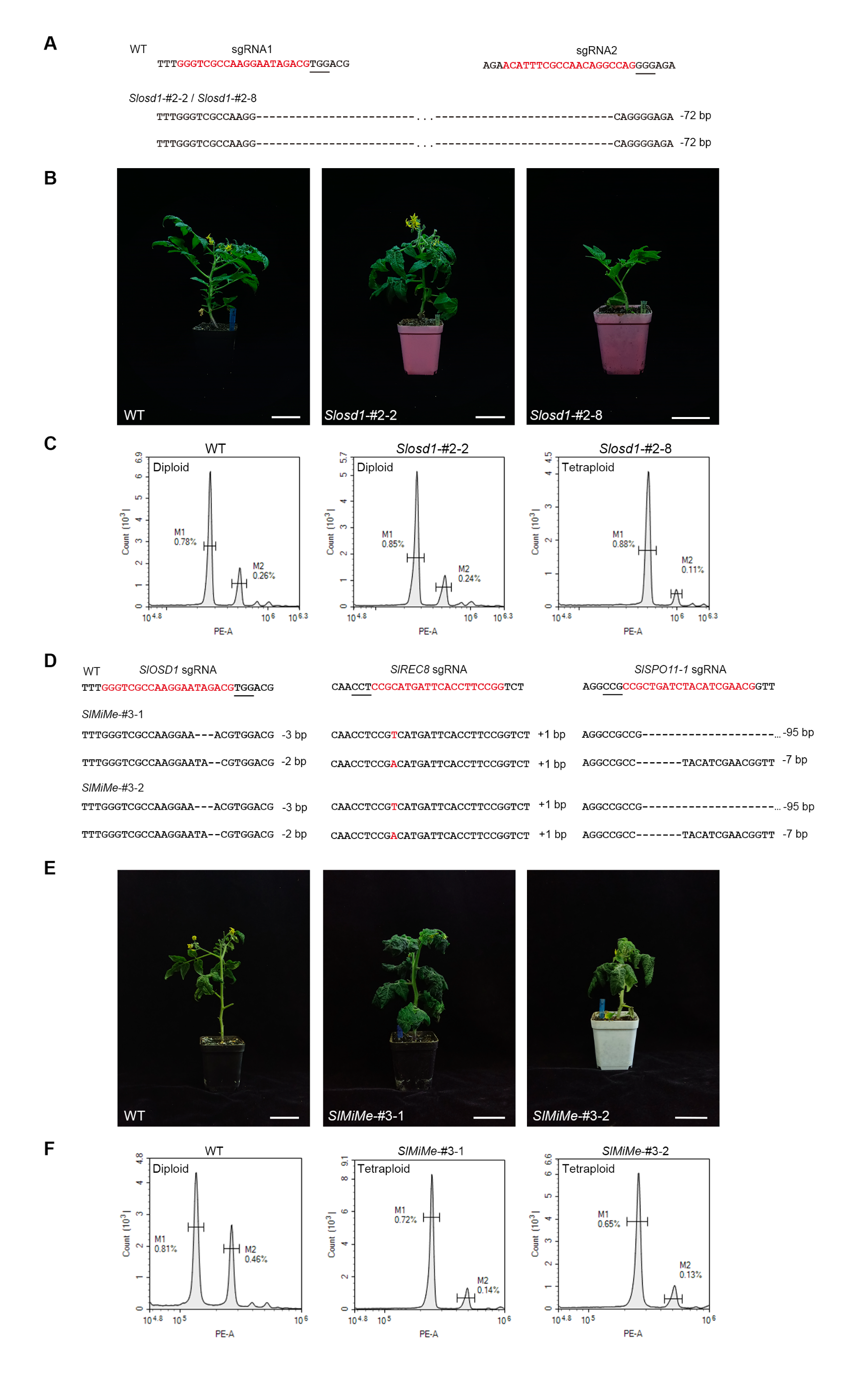


**Fig. S7** Mutation site, phenotype and ploidy detection of *Slosd1*-#2 and *SlMiMe*-#3 T_1_ generation plants. **A** Mutations in *Slosd1*-#2-2 and *Slosd1-*#2-8 at two sgRNA target sites. **B** Phenotype of wild-type plants and representative *Slosd1* mutant plants in the T_1_ generation. Bars, 5 cm. **C** Ploidy analysis of *Slosd1* mutants was analyzed using flow cytometry. Among the 18 *Slosd1* plants, #2-8 was identified as tetraploid, the others were diploid. **D** Mutations in the sgRNA target sites of representative mutants *SlMiMe*-#3-1 and #3-2. **E** Phenotype of wild-type plants and representative *SlMiMe* mutant plants in the T_1_ generation. Bars, 5 cm. **F** The ploidy of the *SlMiMe* representative mutants was analyzed using flow cytometry. Both *SlMiMe*-#3-1 and #3-2 were tetraploid.


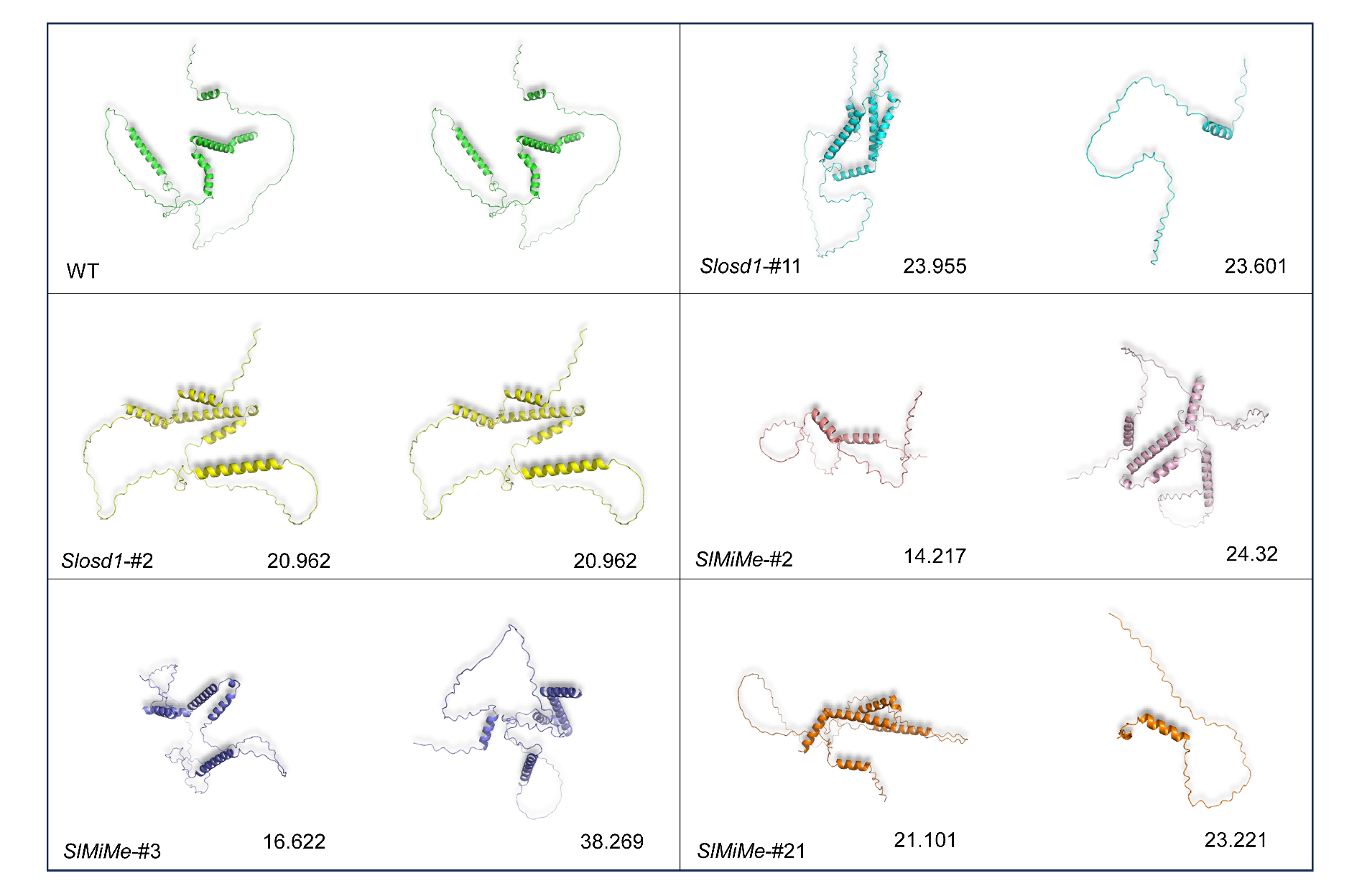


**Fig. S8** Predicted 3D structures of SlOSD1 protein in wild-type, *Slosd1* diploid mutant, and *SlMiMe* diploid mutant plants, generated using AlphaFold 3. Although some of these mutants did not exhibit frameshift mutations, their protein structures still underwent significant changes. Root mean square deviation (RMSD) values, shown in the lower-left corner of the figure were calculated by aligning the three-dimensional structures of the SlOSD1 protein in both mutant and wild-type plants using PyMOL software. A higher RMSD value indicates a greater disparity between protein structures and a lower degree of similarity.
